# Cancer survival stories: Perception, creation, and potential use case

**DOI:** 10.1111/hex.13760

**Published:** 2023-05-03

**Authors:** Claudia Canella, Martin Inderbitzin, Manuela Oehler, Claudia M. Witt, Jürgen Barth

**Affiliations:** ^1^ Institute for Complementary and Integrative Medicine University Hospital Zurich and University of Zurich Zurich Switzerland; ^2^ Institute of Social Medicine, Epidemiology and Health Economics Charité—Universitätsmedizin Berlin, corporate member of Freie Universität Berlin, Humboldt‐Universität zu Berlin, and Berlin Institute of Health Berlin Germany; ^3^ My Survival Story Foundation Zurich Switzerland

**Keywords:** cancer, cancer survivors, citizen science, participatory research, patient narratives, patient education, mixed methods

## Abstract

**Background:**

Cancer patients often search for information about their health conditions online. Cancer patient narratives have established themselves as a way of providing information and education but also as an effective approach to improving coping with the disease.

**Objective:**

We investigated how people affected by cancer perceive cancer patient narratives and whether such stories can potentially improve coping during their own cancer journeys. Additionally, we reflected on whether our co‐creative citizen science approach can contribute to gaining knowledge about cancer survival stories and providing peer support.

**Design, Setting and Stakeholders:**

We applied a co‐creative citizen science approach by using quantitative and qualitative research methods with stakeholders (i.e., cancer patients, their relatives, friends and health professionals).

**Main Outcome Measures:**

Understandability and perceived benefits of cancer survival stories, coping, emotional reactions to the stories and helpful characteristics of the stories.

**Results:**

Cancer survival stories were considered intelligible and beneficial, and they potentially support positive emotions and coping in people affected by cancer. Together with the stakeholders, we identified four main characteristics that evoked positive emotions and that were considered especially helpful: (1) positive attitudes towards life, (2) encouraging cancer journeys, (3) individual coping strategies for everyday challenges and (4) openly shared vulnerabilities.

**Conclusions:**

Cancer survival stories potentially support positive emotions and coping in people affected by cancer. A citizen science approach is suitable for identifying relevant characteristics of cancer survival stories and may become a helpful educational peer support resource for people coping with cancer.

**Patient or Public Contributions:**

We adopted a co‐creative citizen science approach, wherein citizens and researchers were equally involved throughout the entire project.

## 1 BACKGROUND

According to a recent press release by the World Health Organization, there were 19.3 million new cancer cases worldwide in 2020.^1^ These data indicate that one in five people will develop cancer at some point in their lives. Moreover, with an increasing number of effective cancer therapies, the number of cancer survivors (i.e., cancer patients after 5 years of their cancer diagnosis) has been steadily increasing. For example, in 2020, the number of cancer survivors was approximately 50.6 million people.^1^ With this number of cancer survivors, there is a growing awareness of the need for supportive care in cancer care, especially after cancer treatment. Specifically, there are concerns about both, high healthcare costs caused by cancer, and more importantly, the cognitive, physical, emotional and spiritual burdens on the quality of life, productivity and social participation of cancer survivors.^2^, ^3^

Currently, patients search for information about their health condition in a variety of ways, including on the internet, online platforms and social media.^4^, ^5^ Patient narratives have established themselves as a way of providing information and education, as well as an effective approach to improving coping with the disease.^6^, ^7^, ^8^, ^9^, ^10^ An example of an online platform is the Dipex charity.^5^, ^11^ Such platforms have also been established for cancer patients.^12^, ^13^, ^14^, ^15^

In this article, we refer to such cancer patients' narratives as ‘cancer survival stories’. Here, the term survival refers to ways of coping during the cancer journey and not to the survival rates of people with cancer. Drewniak and colleagues^16^ have provided a systematic review of the risks and benefits of web‐based patient narratives, wherein they concluded that they are overall supportive; however, they are also challenging to quantitatively measure. To date, patient narratives have been predominantly investigated by applying qualitative research methods.^5^, ^9^, ^17^

It has been shown that cancer survival stories can effectively inform and educate patients about possible treatments.^8^, ^16^, ^18^, ^19^ An important underlying concept is a peer‐to‐peer concept, where peers are educated to support people affected by cancer based on a trustful relationship and high credibility.^20^, ^21^ Furthermore, cancer survival stories can help patients to better cope with psychological challenges due to cancer, although coping is a complex construct to measure.^4^, ^16^, ^22^ The storytellers' narratives add a personal twist to the content. Compared to impersonal information, cancer survival stories may also elicit stronger positive (as well as negative) affective responses from patients.^4^, ^23^

There are significant reasons to use a citizen science approach in health research. The prioritization of the patients' values and needs is incorporated into the concept of evidence‐based medicine^24^ and has led to the development of increasingly participative approaches in health research.^25^ According to the principles of Citizen Science,^20^ the stakeholders should at the very least have a say and in the best case a right to participate in developing the research process, conducting the analysis, and coauthoring the publication of the results.^25^, ^26^, ^27^, ^28^, ^29^, ^30^ Specifically, health research should incorporate the priorities, values and needs of those individuals affected by a health condition. Citizen science is a suitable approach to apply this claim in practice.^25^, ^26^, ^27^, ^28^, ^29^, ^30^, ^31^

With our study, we wanted to contribute to the existing research about cancer narratives by exploring the perception of such narratives with a highly participative citizen science methodological approach. Therefore, we did not assess clinical outcomes, which could be a focus of an upcoming study based on the findings of this study.

The ‘My Survival Story’ Foundation produces personal narratives of cancer survivors throughout the world and with different types of cancers. The stories are shared online for free in the form of videos and podcasts.^32^ The videos range in length between 3 and 5 min, whereas the podcasts range in length between 15 and 20 min. Most of the stories are in English, and a few stories are in Spanish, Swiss German and Japanese, with all of the stories having English subtitles. In this article, we refer to the stories of the My Survival Story Foundation as ‘my survival stories’.

The aim of the study was to investigate perceptions, potentials and challenges of cancer survival stories. Using quantitative and qualitative research methods, we investigated different interrelated research questions with stakeholders (cancer patients, their relatives, friends and health professionals): (1) With an online survey, we investigated how stakeholders perceive and evaluate ‘my survival story’ narratives. (2) In qualitative interviews, we asked stakeholders how they perceive the narratives, which aspects of the narratives were meaningful to them, and if they could be of help to cope better during their own cancer journeys. (3) In a workshop, we explored ways in which stakeholders could develop and record their own ‘my survival story’ and provide peer support. In addition, we explored if our co‐creative citizen science approach can contribute to gaining transformative knowledge about the ‘my survival stories’, as well as if they have the potential for becoming a resource for patient education.

## 2 METHODS

### 2.1 Co‐creative citizen science approach

We applied a co‐creative participative citizen science approach by using quantitative and qualitative research methods in 2020.^28^, ^29^, ^30^, ^33^, ^34^ Our approach followed the 10 principles described by the European Citizen Science Association^28^ (see Table 1). In addition, we followed the typology of Shirk and colleagues by defining ‘co‐creative’ as the degree of citizen participation in our project,^30^ meaning that at least some citizens are equally involved and responsible as the researchers in every step of the research project.

**Table 1:** Citizen science process, timeline, setting, duration and documentation.

Timeline months in 2020	Topic and data	Setting	Duration^a^ minutes	Documentation	Reference to European Citizen Science Association (ECSA) principles^b^
Feb	Core team and process moderator: kick off workshop	Onsite	120′	Protocol and whiteboard	1, 4
Mar	Workshop core team and process moderator: Shaping the project	Onsite	240′	Protocol and whiteboard	1, 2, 4
Apr	Workshop core team and process moderator: Planning data collection	Online	240′	Protocol	4
Jul	Interviews stakeholders	Online	60′–90′ for each interview	Videotaped	3, 5
Aug	Core team and process moderator: Intersubjective validation analyses interview data. Planning next steps	Online	240′	Protocol	2, 4
Sep	Core team and process moderator: Shaping the stakeholder workshop	Onsite	240′	Protocol and whiteboard	4
Aug–Nov	Survey stakeholders	Online		Survey data	3, 4, 5
Nov	Stakeholder workshop	Online	240′	Videotaped	3, 4, 5
Nov	Core team and process moderator: Intersubjective validation analyses stakeholder workshop	Online	60′	Protocol	2, 4
Nov	Core team: Next steps and science communication strategy	Online	120′	Protocol	3, 4, 5, 6, 7, 8, 9, 10

a Duration indicates the duration of the respective event, excluding preparation, postprocessing and the time invested for data analyses of the individual members of the core team.

b ECSA—Principles 28: 1—Citizen science projects actively involve citizens in the scientific endeavour that generates new knowledge or understanding. Citizens may act as contributors, collaborators or as project leaders and have a meaningful role in the project. 2—Citizen science projects have a genuine science outcome. For example, answering a research question or informing conservation action, management decisions or environmental policy. 3—Both professional scientists and citizen scientists benefit from taking part. Benefits may include the publication of research outputs, learning opportunities, personal enjoyment, social benefits, satisfaction through contributing to scientific evidence, for example, to address local, national and international issues and through that, the potential to influence policy. 4—Citizen scientists may, if they wish, participate in multiple stages of the scientific process. This may include developing the research question, designing the method, gathering and analyzing data and communicating the results. 5—Citizen scientists receive feedback from the project. For example, how their data are being used and what the research, policy or societal outcomes are. 6—Citizen science is considered a research approach like any other, with limitations and biases that should be considered and controlled for. However, unlike traditional research approaches, citizen science provides the opportunity for greater public engagement and democratisation of science. 7—Citizen science project data and metadata are made publicly available and where possible, results are published in an open‐access format. Data sharing may occur during or after the project unless there are security or privacy concerns that prevent this. 8—Citizen scientists are acknowledged in project results and publications. 9—Citizen science programmes are evaluated for their scientific output, data quality, participant experience and wider societal or policy impact. 10—The leaders of citizen science projects take into consideration legal and ethical issues surrounding copyright, intellectual property, data‐sharing agreements, confidentiality, attribution and the environmental impact of any activities.

A core team consisting of one member from the My Survival Story Foundation (Martin Inderbitzin, Citizen) and two members from the University Hospital Zurich (Claudia Canella and Jürgen Barth, researchers) were equally involved during the entire research process. In addition, different stakeholders (health professionals, cancer survivors, relatives and friends) actively participated in the project. An external process moderator supported the whole research process fostering a constructive and power‐balanced collaboration between all stakeholders and the research team.

We used a quantitative online survey to assess the understandability, perceived benefits, emotional reactions to the ‘my survival stories’ and if they are perceived as supportive by a broad range of stakeholders. In parallel, we engaged stakeholders in semistructured qualitative online interviews (find the interview guidelines in Supporting Information: Appendix 1) and in an online co‐creative stakeholder workshop (see Table 1, for an overview of the citizen science approach). Both the interviews and the workshop were video recorded. Moreover, we aimed to deepen our understanding of what content of the ‘my survival stories’ was helpful to the stakeholders and how it related to the personal situations of the stakeholders. In the workshop, we introduced different storytelling formats to the stakeholders and asked for practical hints on how the existing My Survival Story platform could be further developed towards a ‘Do‐It‐Yourself’ platform, where stakeholders could directly upload their stories produced on their own.

In our project, ‘co‐creation’^30^ happened on two levels: (1) within the core team (Martin Inderbitzin, Claudia Canella and Jürgen Barth), and (2) between the core team and the stakeholders creating new content during the workshop.

The core team (Martin Inderbitzin, Claudia Canella and Jürgen Barth) shares an interest in cancer research, as researchers as well as people affected by cancer, and aim to foster coping with cancer. Find further information about the authors in the ‘consolidated criteria for reporting qualitative studies (COREQ): 32‐item checklist’ in Supporting Information: Appendix 2.^35^

### 2.2 Online survey

The aim of the online survey was the evaluation of the videos and podcasts of the My Survival Story platform by different stakeholders. We recruited participants via the Institute's website and newsletter, as well as via the My Survival Story website, newsletter and social media channels. The stakeholders were eligible if they were cancer patients independent of disease status and treatment, cancer survivors or relatives or friends of cancer patients. All participants received similar information in the initial advertisement of the study to become enroled in the survey (via newsletter, Facebook, etc.) and received within the survey additional information about the aim of the project. Data were electronically collected between August and November 2020 via an online survey (SoSci Survey). Within this survey, the stakeholders were allocated to one specific media but had the opportunity to rate more than one if they opted in for another round of the evaluation. The stakeholders were randomly assigned to 1 out of 12 videos or 1 out of 8 podcast episodes.

As baseline data, we assessed sociodemographic variables (e.g., age, gender, cancer diagnosis, year of diagnosis, treatment status vs. relationship to a cancer patient and country of origin) and information media consumption (e.g., being alone while watching media and the device that was used). As outcome data, the evaluation of media encompassed the following four dimensions: the understandability of the media, the perceived benefits (for themselves and for others), coping with difficulties associated with cancer and emotional reactions to the story (relaxed, positive and negative emotions).

Descriptive statistics (e.g., numbers and percentages for categorical and dichotomous variables; means and standard deviations for continuous variables) are reported for the baseline (i.e., sociodemographics, clinical information) and outcome data (i.e., evaluation of the media). The analyses were conducted for all media and stratified according to media and user characteristics (i.e., video vs. podcast, patient vs. relative/friend and match vs. mismatch with the protagonist's gender). All of the analyses were performed by using IBM SPSS 26. All of the analyses were conducted with original data, and missing values were not imputed because the number of missing data per item was very low (<5%).

### 2.3 Qualitative participatory process

In the qualitative participatory process, we followed the method of rapid qualitative evaluation,^36^, ^37^ which we applied to the interviews and the stakeholder workshop. For recruitment, we used a purposive sampling strategy^38^ and recruited patients via the My Survival Story website, newsletter and social media channels. We searched for cancer health professionals, cancer survivors, relatives and friends who were willing to actively engage with the ‘my survival stories’ and engage with their own narratives in a workshop. During recruitment, we prioritized cancer survivors and relatives over health professionals and other representatives, for example, of patient organizations, NGOs, the health industry and politics, because we considered the former as key stakeholders in experiencing the narratives. We included cancer survivors independent of cancer type, disease status and treatment. Furthermore, we aimed at collecting saturated data, that is, a broad spectrum of reported experiences, regarding the evaluation of content, the setting of the ‘my survival stories’ and the expressed relations to the specific situations. We were limited in the recruitment process due to the COVID‐19 pandemic and the duration of the study.^36^, ^38^

The first author (Claudia Canella) created the interview guidelines (find them in Supporting Information: Appendix 1) and conducted the interviews. She is a trained qualitative researcher with 20 years of experience; that is, she conducted and analyzed interviews with about 300 people affected by cancer in the last decade.

To analyze the qualitative data, we used MAXQDA Software (Release 18.2.4) and followed the method of rapid qualitative evaluation^36^, ^37^ by writing research diaries, using spreadsheets to maintain the overview of the data and summarizing the interviews and the workshops. We linked each paragraph of these summaries to the according video recordings and transcribed partially the key statements. We identified the main themes related to our research questions by inductively collapsing all the data from the research diaries, spreadsheets, summaries, videos and transcripts.^36^, ^37^ For each step of the data analysis, we performed an intersubjective validation process between two members of the core team (one researcher and one citizen) to verify the reliability and robustness of the data analysis.^27^, ^38^ Subsequently, we discussed our results with the coauthors, the process moderator, and the participants of the stakeholder workshop.

Find further details about the qualitative research process in the ‘consolidated criteria for reporting qualitative studies (COREQ): 32‐item checklist’ in Supporting Information: Appendix 2.^35^

## 3 RESULTS

In total, 165 stakeholders participated in the project. Their characteristics are shown in Table 2.

**Table 2:** Baseline characteristics of stakeholders.

	Online survey (*n* = 158)	Interviews/workshop (*n* = 7)
Gender identity		
Female	113 (76.4%)	4
Male	34 (22.9%)	3
Other	1 (0.7%)	–
Age (range)	44.42 (20–78)	54 (37–81)
Stakeholder group		
Cancer patients	101 (63.9%)	4
Relatives/friends	41 (25.9%)	4
Health professionals	–	1
Other	6 (3.8%)	–
Cancer diagnoses (clustered)		
Breast/ovarian	64 (43.2%)	1
Prostate/testicular	5 (3.4%)	–
Colon	11 (7.4%)	–
Lung	2 (1.4%)	–
Pancreas	8 (5.4%)	–
Leukaemia	7 (4.7%)	–
Brain	5 (3.4%)	1
Skin	4 (2.7%)	–
Lymphoma	15 (10.1%)	–
Other	–	1
Multiple diagnoses	5 (3.4%)	1
Treatment status^a^		
Under treatment	55 (34.8%)	–
Treatment completed	46 (29.1%)	4
Diagnoses time, years^b^		
≤5	107 (67.7%)	2
>5	30 (19%)	2

a This question was only asked for persons affected by cancer.

^b^ Referring to 2020.

### 3.1 Online survey

**Figure 1:**
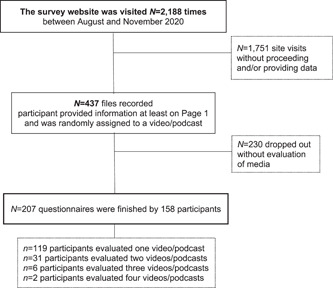
Flowchart recruitment process online survey.

The analysis population consisted of *N* = 158 stakeholders with a total of 207 evaluated media (see Figure 1, for details of the recruitment process). Approximately 25% of the stakeholders viewed more than one video/podcast episode. Stakeholders were mainly from Switzerland (34.5%), the United States of America (23%) and other European countries (except for Switzerland/Germany at 10.8%). Other countries/continents stated as origins were the United Kingdom, Asia, Germany, South America, Canada, Australia, Africa and other countries. Most of the stakeholders (96.1%) watched the video or listened to the podcast when alone, and 89.3% of the stakeholders finished the video/podcast. The device that was most often used as mobile phones (67%), followed by computers (25.6%) and tablets (7.4%).

Overall, all of the media were evaluated as being very positive (see Table 3). Stakeholders found the stories to be easy to understand and convey a clear message. Moreover, they perceived the stories as being beneficial, meaning that they would like to see or hear more similar stories and would recommend these types of stories to other people affected by cancer.

**Table 3:** Evaluation outcomes for the total sample and stratified by gender, involvement and gender‐matched type of media (mean [*M*] and standard deviation [SD]).

Subgroup	Understand‐ability,^a^ *M* (SD)	Perceived benefit,^b^ *M* (SD)	Coping,^c^ *M* (SD)	Emotions,^d^ *M* (SD)		
				Relaxed	Positive emotions	Negative emotions
Total sample	8.94 (1.86)	8.56 (1.98)	8.06 (2.19)	6.87 (2.32)	8.35 (1.82)	2.92 (1.91)
Gender						
Female *n* = 147	8.89 (1.97)	8.57 (2.01)	8.17 (2.15)	6.74 (2.34)	8.43 (1.85)	2.89 (1.87)
Male *n* = 43	9.27 (1.06)	8.80 (1.46)	7.92 (1.99)	7.49 (2.15)	8.17 (1.71)	2.92 (1.95)
Stakeholder						
Patient *n* = 132	8.91 (1.93)	8.56 (1.96)	8.11 (2.18)	6.88 (2.31)	8.29 (1.89)	2.99 (1.89)
Relatives/friend *n* = 51	9.05 (1.85)	8.69 (1.84)	8.06 (2.07)	7.19 (2.33)	8.58 (1.72)	2.71 (1.88)
Gender in media and gender of stakeholder						
Mismatch *n* = 101	8.95 (1.88)	8.57 (1.98)	8.05 (2.93)	6.89 (2.49)	8.24 (1.78)	2.87 (1.86)
Match *n* = 100	8.92 (1.84)	8.55 (1.99)	8.04 (2.10)	6.83 (2.16)	8.45 (1.77)	2.97 (1.96)
Media type						
Video *n* = 145	9.12 (1.80)	8.67 (1.96)	8.07 (2.18)	6.98 (2.28)	8.51 (1.68)	2.89 (1.94)
Podcast *n* = 56	8.46 (1.91)	8.27 (2.02)	7.98 (2.23)	6.57 (2.43)	7.92 (2.11)	2.98 (1.82)

a Instruction: Can you please indicate on a scale from 1 to 10 how much you agree about the following statements? (1 = strongly disagree, 10 = strongly agree). Item 1: The story was easy to understand. Item 2: The story had a clear message.

b Instruction: Can you please indicate on a scale from 1 to 10 how much you agree about the following statements? (1 = strongly disagree, 10 = strongly agree). Dimension helpfulness: Item 1: I would like to see or hear more stories like this one. Item 2: I would recommend these kinds of stories to other cancer patients or people affected by cancer.

c Instruction: Can you please indicate on a scale from 1 to 10 how much you agree about the following statements? (1 = strongly disagree, 10 = strongly agree). Dimension supportive coping: Item 1: The story makes me feel confident that I will find a way to cope with my situation. Item 2: The story motivates me to continue with my treatment or to support somebody in treatment.

^d^ Instruction: How did you feel while watching the movie or listening to the podcast? Can you please indicate on a scale from 1 to 10 how much you experienced the following emotions? (1 = not at all, 10 = very much). Dimension relaxed: Calm; relaxed. Dimension positive emotion: Hopeful; inspired. Dimension negative emotion: Anxious; worrisome; upset; angry.

The stakeholders perceived the stories as being supportive of their coping with their situation and as a motivation for their further cancer care or for further supporting someone receiving cancer care. As an emotional reaction, the consumption of media was perceived as being moderately relaxing (calm/relaxed) but evoked strong positive emotions (hopeful/inspired). Correspondingly, negative emotions when consuming the media were rated as being rather low (stressed/worried/anxious/angry).

We compared the perceptions of the survey participants, as shown in the lower portion of Table 3. The evaluation of the media did not differ in the subgroups with specific participants' characteristics or media types. This means that media were similarly evaluated across gender, stakeholder groups and types of media. Additionally, the similarity to the protagonist in terms of gender (match) did not make any difference in the evaluation of the media.

### 3.2 Qualitative interviews

We conducted four qualitative semistructured face‐to‐face online interviews and one focus group with three family members. Two of these family members were also cancer survivors. That is why they are listed in Table 2 as belonging to both stakeholder groups, family members and cancer survivors. In total, we interviewed four cancer survivors, three family members, one friend of several cancer survivors and a cancer nurse.

Quotes were originally in Swiss German and German and were translated into English by the authors. The quotes are reported indented, and between quotation marks in the following text. More related quotes can be found in Table 4. The initials are pseudonyms and the numbers indicate to the position in the transcript.

**Table 4:** Representative quotes from the stakeholders.

Topic	Representative quotes^a^
Positive attitudes towards life	‘…The winner is not the one who complains because he has cancer, but the one who makes the best out of it’. (CS MSS_Interview MS_20200721, pos. 14)
Encouraging cancer journeys	‘…to show that he can do that, is allowed to do that and likes to do that, riding a bike and going camping…that is probably why he did not just ride his bike around the lake in Germany, but went to Alaska ‐ to show, I can do that’. (CS MSS_Interview MS_20200721, pos. 18)
Individual coping strategies for everyday challenges	‘I thought it was very nice that the family and the children were also shown in this film…A topic that one often encounters in everyday life…also in my professional environment:…how can one talk to children about cancer?…that was vivid and well done in the film…’ (CS MSS_Interview SB_20200720, pos. 7)
Openly shared vulnerabilities	‘…that you can and should show emotions in life…otherwise you will explode at some point and become depressed. You just must be the way you are…and that not everything is so great [just] because you survived; that there are also phases of sadness in life…or mood swings. That is part of life….that you also express that…’ (CS MSS_Interview SW_20200717, pos. 64)

a These quotes were translated into English by the authors. The interviews were conducted in Swiss German (1), German (2) and in mixed language Swiss German/English (1). Initials are pseudonyms; numbers indicate line numbers in the respective transcript.

The stakeholders indicated that the ‘my survival stories’ were ‘intelligible’ to them, but they would wish to listen or watch ‘more stories in their native language’, rather than solely in English.

A lot of our patients do not understand English. Frequent native languages in our patients are: Italian, Portuguese, Spanish, Croatian, Serbian, Turkish, Russian…Perhaps there are also cultural differences in the perception of such videos. I suspect there are differences in perception, processing, meaning, handling. It would be best then to have videos from people from the same cultural environment. (CS MSS_Interview SB_20200720, Pos. 72‐73)

The focus, form, length and aesthetics of ‘my survival stories’ were much appreciated by the stakeholders.

The stakeholders supported both media formats (audio and video). Although most of the stakeholders preferred video because they could ‘visually relate to the storytellers’, some stakeholders preferred audio because it provides an ‘open space for their own imagination’.

When I listen to the podcast, it creates an image in my head and that's why it's intimate and close. It's a part of me, I helped create the story, I'm part of the story. In the video, I don't have that. (CS MSS_Interview MS_20200721, pos. 90)

Most of the stakeholders preferred to watch or listen to ‘my survival stories’ alone in their homes. However, they also highlighted that the preferences regarding where, when, and with whom to listen to the stories are highly individualized.

The time at which the stakeholders started to listen to ‘my survival stories’ during their own cancer journey depended on when they were first informed about the existence of this platform. Some patients started listening to the stories shortly after their diagnoses, and other patients began listening during their cancer treatment, after cancer treatment and during the survivor stage. Stakeholders highlighted that they believed that there is ‘no common, most suitable point in time during the cancer journey’ to listen to ‘my survival stories’, as it is ‘highly individualized’ when someone benefits the most from the stories.

We identified four main characteristics of the ‘my survival stories’ that stakeholders considered to be especially helpful (please find related quotes from the interviews in Table 4):

1. positive attitudes towards life,
2. encouraging cancer journeys,
3. individual coping strategies for everyday challenges and
4. openly shared vulnerabilities.

In general, the stakeholders related to ‘my survival stories’ in terms of a ‘positive attitude towards life’ and their cancer journey, which was reinforced by the ‘encouraging cancer journeys’ of the protagonists in ‘my survival stories’. The stakeholders appreciated stories that demonstrated coping styles and ‘individual solutions to everyday problems’. However, the stakeholders also appreciated the ‘openly shared vulnerabilities’ of the protagonists.

The detachment from specific cancer types and therapies of the ‘my survival stories’ enabled the stakeholders to relate to topics that most people affected by cancer are confronted with; for example, topics regarding ‘how to tell the children’ or ‘how to return to normal life’ after the cancer treatment.

Most of the stakeholders highlighted that everyone affected by cancer is an ‘individual with different needs at different moments’. Therefore, stakeholders opted for a need‐oriented approach for future new stories that would be recorded. They also wished for stories to be recorded in different languages, stories recorded from the perspectives of relatives or friends, and more stories about individual coping strategies for everyday challenges.

Some of the stakeholders considered stories of palliative or end‐of‐life situations to be potentially problematic when asked about what type of new stories should not be produced in the future. They also believed that the ‘my survival stories’ are not suitable for persons who have lost loved ones to cancer or who are in palliative or end‐of‐life situations. In addition, some stakeholders, such as the cancer nurse, argued that it could be problematic to show the ‘very lucky stories of survivors’, wherein ‘cancer patients with very low life expectancies lived happily ever after’.

That's what bothered me more about the other videos, the best possible courses with little hope. I feel sorry for the many patients for whom it is exactly the opposite; they have a lot of hope but poor courses. (CS MSS_Interview SB_20200720, pos. 24)

They discussed that such stories could ‘cause false expectations’ in people watching these videos or could worsen the depression or grief of persons who are in end‐of‐life situations or who have lost loved ones to cancer.

### 3.3 Co‐creative workshop

Stakeholders were enthusiastic to share their own stories and experiences. For this reason, we conducted an online workshop with three interested cancer survivors and one friend from the interview stakeholders, wherein they recorded their own stories by using their smartphones.

With the workshop, we explored ways how stakeholders could develop and record their own ‘my survival story’ and upload it to a potential ‘Do‐It‐Yourself‐My Survival Story platform’. We aimed to engage participants with short storytelling‐ and technical instruction videos, as well as with low‐tech smartphone recordings. The stakeholders recorded their stories twice. First, they were only given a general recording timeframe of 5 min at maximum without any further indications (except the instruction to choose a challenging situation during their cancer journey for which they found a good coping strategy). Second, they were provided two short instructions about technical settings, such as light, background, audio and examples of storytelling formats, such as the ‘heroes' journey’, ‘anecdote’ or ‘three act story’ (see, e.g., Field^39^). From our point of view, the stakeholders' second recordings increased in quality related to the technical setting and structure of the storylines.

When asked about their feedback at the end of the workshop, stakeholders would have wished for a more intense exchange amongst the other stakeholders about their stories and would have preferred a feedback loop before publishing a recorded story. As all of the interviewees highlighted that the focus on a positive attitude towards life and encouraging coping strategies are crucial to the ‘my survival stories’, the workshop recordings may have benefited from clearer instruction towards these topics, as the stories from the workshop participants often focused on diagnoses and treatment journeys more than coping mechanisms.

Moreover, we felt that a Do‐It‐Yourself platform would be technically feasible but that the recorded stories would still benefit from feedback to ensure that the main characteristics of the ‘my survival stories’ are preserved. Currently, it is a challenge to provide the resources for providing feedback to Do‐It‐Yourself stories uploaded to the platform.

### 3.4 External process moderation

An external process moderator supported the core team in establishing a constructive and power‐balanced collaboration between all the stakeholders (interviewees, workshop participants, citizens) and the research team. The process moderator was present during the workshops with the core team as well as during the co‐creative workshop with other stakeholders. The moderators' role was evident in the following processes: the establishment of a common language between citizens and researchers, the establishment of working communication channels, reflection on the different working cultures, explication of ‘do's’ and ‘don'ts’ in collaboration, definitions of resources and responsibilities, management of time, documentation of the process and creation of an environment in which team members can optimally contribute their own expertise and creativity.

### 3.5 Science communication

As science communication is an essential part of citizen science, we were committed to reporting on our ongoing progress to the public via different channels (websites and social media channels of the University Hospital Zurich and the My Survival Story Foundation^40^, ^41^).

## 4 DISCUSSION

Cancer survival stories were considered intelligible and beneficial, and they may potentially support positive emotions and cope in people affected by cancer, independent of stakeholder characteristics and media formats. We identified four main characteristics of the stories that evoked these positive emotions and that the stakeholders considered to be especially helpful: (1) positive attitudes towards life, (2) encouraging cancer journeys, (3) individual coping strategies for everyday challenges and (4) openly shared vulnerabilities.

With the growing number of cancer survivors^1^ and the subsequent need for supportive cancer care,^2^, ^3^ cancer survival stories can be one option towards providing information and education, as well as improving coping with the disease. Our study contributed to gaining transformative knowledge about ‘my survival stories’ by applying a co‐creative citizen science approach.

The identified characteristics of the ‘my survival stories’ may be transferable to narratives of other illnesses. It has previously been shown that listening to or watching the experiences of peers can support people in their own journeys across a variety of illnesses.^6^, ^42^ The importance of such peer support is also visible in the growing number of freely accessible peer platforms from governments, international research groups and private initiatives.^11^, ^20^, ^43^, ^44^ In addition, patients' narratives can also help clinicians better understand their patients' experiences and needs.^33^, ^45^, ^46^

In general, a challenge for future cancer survival stories and digital health tools is their sustainability.^8^, ^47^, ^48^ They need to be curated content‐wise towards changing settings, cancer treatments and cultural and social developments.^8^, ^47^, ^48^ In addition, the stories are time‐ and culture‐sensitive^8^, ^47^, ^48^; thus, it would be best to constantly add new stories in different languages, from different cultures, and from different stakeholder groups. To achieve this goal, sustainable human and financial resources are needed.^8^, ^47^, ^48^ Currently, with fast‐evolving technology requiring constant system adaptations, the technical sustainability of online platforms is an additional challenge, as is financial sustainability.^8^, ^47^, ^48^ In contrast, many online platforms are free and easily accessible for most people, which makes them a potentially easily applicable tool for patient information and education.^4^, ^8^, ^16^, ^18^, ^19^

The application of a co‐creative citizen science approach helped to identify the relevant characteristics of the cancer survival stories. It provided us with a needs‐oriented approach to the further development of these types of stories. The stories could be a helpful patient education resource for coping with the illness situation, as we now have a better understanding of what characteristics from the cancer survival stories we can focus on.^4^, ^7^, ^16^, ^49^ In addition, we now know that stakeholders consider the cancer survival stories potentially unsuitable for persons in palliative or end‐of‐life situations, as well as for relatives who have lost loved ones to cancer. However, in our survey, participants reported strong negative emotions neither in general nor towards a specific story.

Our next research step may be to test the cancer survival stories in a patient education setting, such as eLearning. Currently, there is still a question as to whether creating one's own narrative is more effective in coping with one's own situation than ‘only’ consuming other people's narratives.^50^, ^51^, ^52^

### 4.1 Strengths

There were several strengths to our study. The adoption of combining a quantitative online survey with qualitative interviews and a co‐creative stakeholder workshop allowed us to investigate what types of emotions are evoked by cancer survival stories in the stakeholders, as well as which content of the stories are considered to support the people affected by cancer in their own journeys.

### 4.2 Limitations

There were several limitations to our study. We included a limited number of stakeholders in the participatory part of the project. This was mainly due to the outbreak of the COVID‐19 pandemic, wherein people affected by cancer were especially vulnerable, and health professionals were considerably burdened. In addition, a lockdown and a temporary stop of research with vulnerable participants (such as cancer patients) in the hospital made recruiting challenging. However, more diverse stakeholders representing more different stakeholder groups may have resulted in additional insights into relevant characteristics, as well as to further needs towards future cancer survival stories. When concerning the online survey, a comparison between the single stories is limited because of the lack of power. More evaluations of single stories might potentially have indicated differences between specific characteristics of the stories as well as the specific way they were narrated. Our sample was not representative related to the general population affected by cancer (e.g., gender identity, education, age and cancer type), as we mainly recruited participants through our social media channels and likely connected with participants who were open and positive‐minded towards the ‘my survival stories’. Including more people affected by cancer and specifically including negative‐minded people in ‘my survival stories’ may have resulted in additional insights into relevant characteristics, as well as needs and challenges for future cancer survival stories. In general, the recruitment of stakeholders was limited to those individuals understanding English well enough to follow the ‘my survival stories’ via video or podcast. Providing ‘my survival stories’ in more different languages would make the stories accessible to more people affected by cancer.

## 5 CONCLUSION

Cancer survival stories potentially support positive emotions and coping in people affected by cancer, independent of stakeholder characteristics and media formats. Cancer survival stories may be a feasible additional peer support source for cancer patient information and education. A citizen science approach is suitable to identify the relevant characteristics of cancer survival stories that could support people affected by cancer during their cancer journeys.

## AUTHOR CONTRIBUTIONS

Claudia Canella, Martin Inderbitzin and Jürgen Barth were equally involved throughout the entire research project, from designing the research to data collection and analyses and science communication. Claudia M. Witt was actively involved in designing the research project, and Manuela Oehler was actively involved in data analyses of the online survey. Claudia Canella, Manuela Oehler and Jürgen Barth drafted and revised the manuscript. Martin Inderbitzin and Claudia M. Witt critically commented on the manuscript. All of the authors contributed to the interpretation of the results and approved the final version of the manuscript.

## CONFLICT OF INTEREST STATEMENT

Martin Inderbitzin is the founder of the My Survival Story Foundation. The remaining authors declare no conflict of interest.

## ETHICS STATEMENT

The authors submitted the study protocol to the Ethics Committee of Zurich, Switzerland, and, after review, they stated that the study did not fall under the regulation of the Human Research Act of Switzerland (BASEC‐Nr. Req‐2019‐01183). They obtained written informed consent for participation and scientific publication from all of the stakeholders in the qualitative study. Stakeholders in the survey agreed to participate in an online fashion.

## Supporting information

Supporting information.

Supporting information.

## Data Availability

The data that support the findings of this study are available on request from the corresponding author. The data are not publicly available due to privacy or ethical restrictions.
