# Supplementary material for: Cancer survival stories: Perception, creation, and potential use case
Source: Health Expect. 2023 May 3;26(4):1551–61. doi: 10.1111/hex.13760 (PMC10349243; doi:10.1111/hex.13760)
Supplement: Supplementary file 1 — Supporting information. [file HEX-26--s001.docx]

**Appendix 1: Interviewguidelines**These interview guidelines were translated into English by the authors. The interviews were conducted in the native languages of the interviewees; Swiss German, German and one in mixed languages, Swiss German and English.

**Title: Cancer survival stories: perception, creation, and potential use case**

**Authors**

Claudia Canella^1, 2^, Martin Inderbitzin^3^, Manuela Oehler^1^, Claudia M. Witt^2^, Jürgen Barth^1^

1 Institute for Complementary and Integrative Medicine, University Hospital Zurich and University of Zurich, Zurich, Switzerland

2 Charité – Universitätsmedizin Berlin, corporate member of Freie Universität Berlin, Humboldt-Universität zu Berlin, and Berlin Institute of Health, Institute of Social Medicine, Epidemiology and Health Economics, Berlin, Germany

3 My Survival Story Foundation, Zurich, Switzerland

**Interviewguideline cancer patients/survivors**

Introduction: Short recap of the three my survival story videos watched (approx. 5')

*First, let's talk about how you personally experienced watching the stories.*

*To do this, the first thing we would do now is watch one of the three stories you selected together again.
→ Which story would you now like to see again?*

Questions

1. What are your current spontaneous thoughts about this video? (10')
   → if you think back to the moment when you watched this video for the first time: did you have any additional first thoughts than right now?

- *Follow-up questions to differentiate between the videos:*What differences did you see between the three videos you selected?

1. If you think back to the moment when you first watched this video: What touched you most about the story? Why? (5-10')
   → What were your first spontaneous feelings when watching the video?
   → What feelings do you have about this video right now?

- *Follow-up questions to differentiate between the videos:*
  What differences did you see between the three videos you selected?

1. Was there one or more moments in this video that reminded you of your own situation? If yes, which ones and in what way? (5-10')
   → Is there anything from the video that supports you in your situation? If yes, what and in what way?
   → Is there anything that made it rather more difficult for you to deal with your situation? If yes, what and to what extent?
   → *Follow-up questions to differentiate between the three selected videos.*
2. At what point in your personal disease progression do you think you would have most liked to hear these stories? (5')
   → When would you have needed the stories most?
   → What times would have been inconvenient?
   → What is your assessment: which times in the course of cancer patients' illness do you generally consider favorable for listening to such stories?
   → Which points in time do you generally consider rather unfavorable?
3. In what setting do you think you would most like to hear these stories? (5')
   So, for example, alone at home, together with the family or during chemotherapy or on the train, bus or streetcar...
   → Which environment would be unfavorable?
   → What is your assessment: what environment do you generally consider favorable for listening to such stories?
   → Which environment do you generally consider rather unfavorable?
4. How do you find the form of the videos to tell such a story? (5')
   → what other forms would you also like? E.g. audio, textbook, audiobook, social media like Facebook, Instagram etc...
   → Could you imagine yourself telling your story in one of the forms discussed? If yes, which form would you prefer? If no, why not?
5. What topics would you like to learn more about in the form of stories like these? (5-10')
   → Which topics did you miss?
   → What is your assessment: Which topics should be discussed in the videos to support those affected in their situation?
   → In your estimation, which topics should generally not be covered in such videos?

Conclusion of the interview (5')

- Now, is there anything else that you would like to add or that we have not talked about yet that you think is important to mention?

**Interview guideline relatives/friends**

Introduction: Short recap of the three my survival story videos watched (approx. 5').

*First, let's talk about how you personally experienced watching the stories.*

*To do this, the first thing we would do now is watch one of the three stories you selected together again.
→ Which story would you now like to see again?*

Questions

1. What are your current spontaneous thoughts about this video? (10')
   → if you think back to the moment when you watched this video for the first time: did you have any additional first thoughts than right now?

- *If necessary, ask for differentiation between the individual videos:*What differences did you find between the three videos you selected?

1. If you think back to the moment when you first watched this video: What touched you most about the story? Why? (5-10')
   → What were your first spontaneous feelings when watching the video?
   → What feelings do you have about this video right now?

- *If necessary, ask for differentiation between the individual videos:*
  What differences did you find between the three videos you selected?

1. Was there one or more moments in this video that reminded you of your own situation as a family member? If yes, which ones and in what way? (5-10')
   → Is there anything from the video that supports you in your situation? If yes what and to what extent?
   → Is there anything that made it rather more difficult for you to deal with your situation? If yes, what and to what extent?
   → *Follow-up questions to differentiate between the three selected videos.*
2. At what point in your loved one's cancer journey do you think you would have most liked to hear these stories? (5')
   → When would you have needed the stories most?
   → What times would have been inconvenient?
   → What is your assessment: which times in the course of the illness of cancer patients* do you generally consider favorable for listening to such stories?
   → Which points in time do you generally consider to be rather unfavorable?
3. In what setting do you think you would most like to hear these stories? (5')
   So, for example, alone at home, together with the family or during chemotherapy or on the train, bus or streetcar...
   → Which environment would be unfavorable?
   → What is your assessment: what environment do you generally consider favorable for listening to such stories?
   → Which environment do you generally consider rather unfavorable?
4. How do you find the form of the videos to tell such a story? (5')
   → What other forms would you also like? E.g. audio, textbook, audiobook, social media like facebook, Instagram etc...
   → Could you imagine yourself telling your story in one of the forms discussed? If yes, which form would you prefer? If no, why not?
5. About which topics would you like to learn more in the form of such stories? (5-10') (e.g. stories from relatives?)
   → What is your assessment: Which topics should be discussed in the videos to support those affected in their situation?
   → In your estimation, which topics should generally not be discussed in such videos?

Conclusion of the interview (5')

- Now, is there anything else that you would like to add or that we have not talked about yet that you think is important to mention?

**Interviewguideline health professionals**

Introduction: Short recap of the three my survival videos watched (approx. 10').

*First, let's talk about how you personally experienced watching the stories.*

*To do this, the first thing we would do now is watch one of the three stories you selected together again.
→ Which story would you now like to see again?*

Questions

1. What are your current spontaneous thoughts about this video? (10')
   → If you think back to the moment when you watched this video for the first time: did you have any additional first thoughts than right now?

- *Follow-up questions to differentiate between the videos:*What differences did you see between the three videos you selected?

1. If you think back to the moment when you first watched this video: What touched you most about the story? Why? (5-10')
   → What were your first spontaneous feelings when watching the video?
   → What feelings do they have right now about this video?
   → *Follow-up questions to differentiate between the videos:*
    What differences were there for you between the three videos you selected?
2. What is your assessment: Is there anything from the videos you have seen where you think this could support cancer patients in their situation? If yes, what and in what way? (5-10')
   → Is there anything that might make it more difficult for the patients to deal with their situation? If yes, what and to what extent?
   → *Differentiation: What is your assessment of this for other affected persons, e.g. relatives?*
3. Is there anything from the videos you have seen that could support you in dealing with your cancer patients? If yes, what and in what way?
   → Is there anything that could make it more difficult for you to deal with your cancer patients? If yes, what and to what extent?
4. What times in the course of cancer patients' illness do you generally consider favorable for listening to such stories? (5')
   → When do you think patients need such stories the most?
   → Which times do you generally consider to be rather unfavorable?
   → *Differentiation: What is your assessment of this for other affected persons, e.g. relatives?*
5. In general, what environment do you consider conducive to listening to such stories? (5')
   So e.g. alone at home, together with the family or during chemotherapy or on the train, bus or streetcar...
   → Which environment do you generally consider rather unfavorable?
   → *Differentiation: What is your assessment of this for other affected persons, e.g. relatives?*
6. What is your assessment: could such stories be usefully employed in your clinic setting for cancer patients? (5')
   If yes, where and how? If no, why not?
   → *Differentiation: What is your assessment of this for other affected persons, e.g. relatives?*
7. How do you find the form of video to share such a story? (5')
   → What other forms would you also like? E.g. audio, textbook, audiobook, social media like Facebook, Instagram etc...
   → Could you imagine yourself sharing your story in one of the forms discussed? If Yes, which form would you prefer?
    If no, why not?
8. What topics would you like to learn more about in the form of stories like these? (5-10')
   → What topics did you miss?
   → Would you like to hear a story of a professional colleague in dealing with cancer patients or relatives?
   → What is your assessment: Which topics should be discussed in the videos to support those affected in their situation?
   → In your estimation, which topics should generally not be covered in such videos?

Conclusion of the interview (5')

- Now, is there anything else that you would like to add or that we have not talked about yet that you think is important to mention?
