# Supplementary material for: Cancer survival stories: Perception, creation, and potential use case
Source: Health Expect. 2023 May 3;26(4):1551–61. doi: 10.1111/hex.13760 (PMC10349243; doi:10.1111/hex.13760)
Supplement: Supplementary file 2 — Supporting information. [file HEX-26--s002.docx]

**Appendix 2: Consolidated criteria for reporting qualitative studies (COREQ): 32-item checklist**

**Title: Cancer survival stories: perception, creation, and potential use case**

**Authors**

Claudia Canella^1, 2^, Martin Inderbitzin^3^, Manuela Oehler^1^, Claudia M. Witt^2^, Jürgen Barth^1^

1 Institute for Complementary and Integrative Medicine, University Hospital Zurich and University of Zurich, Zurich, Switzerland

2 Charité – Universitätsmedizin Berlin, corporate member of Freie Universität Berlin, Humboldt-Universität zu Berlin, and Berlin Institute of Health, Institute of Social Medicine, Epidemiology and Health Economics, Berlin, Germany

3 My Survival Story Foundation, Zurich, Switzerland

| **No** | **Item** | **Guide questions/description** | **Reporting** |
| --- | --- | --- | --- |
| **Domain 1: Research team and reflexivity** |  |  |  |
| Personal Characteristics |  |  |  |
| 1. | Interviewer/facilitator | Which author/s conducted the interview or focus group? | Interviews: Claudia Canella  Workshops: Claudia Canella, Martin Inderbitzin, Jürgen Barth |
| 2. | Credentials | What were the researcher's credentials? *E.g. PhD, MD* | Claudia Canella: MA  Martin Inderbitzin: PhD  Manuela Oehler: MSc  Jürgen Barth: PhD  Claudia M. Witt: Prof. Dr. med., MBA |
| 3. | Occupation | What was their occupation at the time of the study? | Claudia Canella: Researcher at affiliation 1; Doctoral student at affiliation 2  Martin Inderbitzin: Founder of the My Survival Story Foundation  Manuela Oehler: Researcher at affiliation 1  Jürgen Barth: Head of research at affiliation 1  Claudia M. Witt: Head of working group integrative medicine and digital health at affiliation 2 |
| 4. | Gender | Was the researcher male or female? | Claudia Canella: Female  Martin Inderbitzin: Male  Manuela Oehler: Female  Jürgen Barth: Male  Claudia M. Witt: Female |
| 5. | Experience and training | What experience or training did the researcher have? | Claudia Canella: MA in Cultural Anthropology, GCP trained  Martin Inderbitzin: PhD in Neuropsychology  Manuela Oehler: MSc in Psychology, GCP trained  Jürgen Barth: PhD in Psychology, GCP trained  Claudia M. Witt: Prof. Dr. med., MBA (Epidemiology); GCP trained |
| Relationship with participants |  |  |  |
| 6. | Relationship established | Was a relationship established prior to study commencement? | No |
| 7. | Participant knowledge of the interviewer | What did the participants know about the researcher? e*.g. personal goals, reasons for doing the research* | - What can be found on the institutional websites of affiliations 1, 2, and 3 |
| 8. | Interviewer characteristics | What characteristics were reported about the interviewer/facilitator? e.g. *Bias, assumptions, reasons and interests in the research topic* | - What can be found on the institutional websites of affiliations 1, 2, and 3 and our personal interests in the research topic - At the beginning of the workshop, everybody introduced her-/himself by sharing a hidden talent - Personal interests of the authors in the research topic |
| **Domain 2: study design** |  |  |  |
| Theoretical framework |  |  |  |
| 9. | Methodological orientation and Theory | What methodological orientation was stated to underpin the study? *e.g. grounded theory, discourse analysis, ethnography, phenomenology, content analysis* | - See chapter “Methods – Co-creative citizen science approach”, paragraphs 1, and 3 - See chapter “Online survey” - See chapter “Qualitative participatory process” |
| Participant selection |  |  |  |
| 10. | Sampling | How were participants selected? *e.g. purposive, convenience, consecutive, snowball* | - See chapter “Methods -“Online survey”, paragraphs 1, and 3 - See chapter “Methods – Qualitative participatory process, paragraph 1 |
| 11. | Method of approach | How were participants approached? e*.g. face-to-face, telephone, mail, email* | - See chapter “Methods -“Online survey”, paragraphs 1, and 3 - See chapter “Methods – Qualitative participatory process, paragraph 1, approached by email and telephone |
| 12. | Sample size | How many participants were in the study? | See table 2 |
| 13. | Non-participation | How many people refused to participate or dropped out? Reasons? | - See Figure 1 for the online survey - No dropout, but 10 people refused for the qualitative participatory process without indicating further reasons |
| Setting |  |  |  |
| 14. | Setting of data collection | Where was the data collected? e*.g. home, clinic, workplace* | - All data was collected online |
| 15. | Presence of non-participants | Was anyone else present besides the participants and researchers? | No |
| 16. | Description of sample | What are the important characteristics of the sample? *e.g. demographic data, date* | See table 2 |
| Data collection |  |  |  |
| 17. | Interview guide | Were questions, prompts, guides provided by the authors? Was it pilot tested? | See appendix 1 and table 1 |
| 18. | Repeat interviews | Were repeat interviews carried out? If yes, how many? | No |
| 19. | Audio/visual recording | Did the research use audio or visual recording to collect the data? | Yes, see table 1 |
| 20. | Field notes | Were field notes made during and/or after the interview or focus group? | Yes |
| 21. | Duration | What was the duration of the interviews or focus group? | See table 1 |
| 22. | Data saturation | Was data saturation discussed? | Yes, see chapter “Methods – Qualitative participatory process, paragraph 1 |
| 23. | Transcripts returned | Were transcripts returned to participants for comment and/or correction? | No, but the results from the interviews were discussed within the core team, with the process moderator, the co-authors as well as with the participants of the workshop |
| **Domain 3: analysis and findings**z |  |  |  |
| Data analysis |  |  |  |
| 24. | Number of data coders | How many data coders coded the data? | n.a. |
| 25. | Description of the coding tree | Did authors provide a description of the coding tree? | n.a. |
| 26. | Derivation of themes | Were themes identified in advance or derived from the data? | Themes were derived from the data; see chapter “Methods – Qualitative participatory process, paragraph 2 |
| 27. | Software | What software, if applicable, was used to manage the data? | - Qualitative data: MAXQDA Software (Release 18.2.4) - Quantitative data: IBM SPSS 26 |
| 28. | Participant checking | Did participants provide feedback on the findings? | - Yes, the results from the interviews were discussed within the core team, with the process moderator, the co-authors as well as with the participants of the workshop - No feedback for the results of the online survey |
| Reporting |  |  |  |
| 29. | Quotations presented | Were participant quotations presented to illustrate the themes / findings? Was each quotation identified? e*.g. participant number* | See table 4 and chapter “Results – Qualitative interviews”, paragraph 2 |
| 30. | Data and findings consistent | Was there consistency between the data presented and the findings? | See tables 3 and 4 and chapter “results” |
| 31. | Clarity of major themes | Were major themes clearly presented in the findings? | See tables 3 and 4 and chapter “results” |
| 32. | Clarity of minor themes | Is there a description of diverse cases or discussion of minor themes? | Yes, see chapter “Results – Qualitative interviews”, paragraphs 4, 6, and 11 |

Allison Tong, Peter Sainsbury, Jonathan Craig, Consolidated criteria for reporting qualitative research (COREQ): a 32-item checklist for interviews and focus groups, International Journal for Quality in Health Care, Volume 19, Issue 6, December 2007, Pages 349–357, <https://doi.org/10.1093/intqhc/mzm042>
